# A Novel Large-Scale Deletion of The Mitochondrial DNA of Spermatozoa of Men in North Iran

**DOI:** 10.22074/ijfs.2015.4185

**Published:** 2015-02-07

**Authors:** Maryam Gholinezhad Chari, Abasalt Hosseinzadeh Colagar, Ali Bidmeshkipour

**Affiliations:** 1 Fatemehzahra Infertility and Reproductive Health Research Center, Babol University of Medical Sciences, Babol, Iran; 2 Department of Biology, Faculty of Basic Sciences, Razi University, Kermanshah, Iran; 3 Department of Biology, Faculty of Basic Sciences, University of Mazandaran, Babolsar, Iran

**Keywords:** Mitochondrial DNA, Large Deletions, Sperm Motility

## Abstract

**Background:**

To investigate the level of correlation between large-scale deletions of the mitochondrial DNA (mtDNA) with defective sperm function.

**Materials and Methods:**

In this analytic study, a total of 25 semen samples of the nor- mozoospermic infertile men from North of Iran were collected from the IVF center in an infertility clinic. The swim-up procedure was performed for the separation of spermatozoa into two groups; (normal motility group and abnormal motility group) by 2.0 ml of Ham’s F-10 medium and 1.0 ml of semen. After total DNA extraction, a long-range polymerase chain reaction (PCR) technique was used to determine the mtDNA deletions in human spermatozoa.

**Results:**

The products of PCR analysis showed a common 4977 bp deletion and a novel 4866 bp deletion (flanked by a seven-nucleotide direct repeat of 5΄-ACCCCCT-3΄ within the deleted area) from the mtDNA of spermatozoa in both groups. However, the frequency of mtDNA deletions in abnormal motility group was significantly higher than the normal motility group (56, and 24% for 4866 bp-deleted mtDNA and, 52, and 28% for 4977 bp-deleted mtDNA, respectively).

**Conclusion:**

It is suggested that large-scale deletions of the mtDNA is associated with poor sperm motility and may be a causative factor in the decline of fertility in men.

## Introduction

Sperm motility is one of the key indicators of fertility in men. Spermatozoa require enormous amount of energy for their survival and fast speed of flagella during fertilization (1, 2). There are ~22-80 mitochondria in the midpiece of a single mature mammalian spermatozoon (2-4). Mitochondria facilitate sperm’s rigorous demand for energy through oxidative phosphorylation (OXPHOS) via the electron transport chain (ETC) in eukaryotic cells. This process is accomplished by the respiratory chain and ATP synthesis, which comprise a series of protein complexes that are encoded by both nuclear and mitochondrial genomes (nDNA and mtDNA respectively) (2, 4). Mitochondria possess their own unique genome, which is compartmentalized away from the nDNA. Human mtDNA is a 16569 base pair double-stranded circular DNA molecule that codes 13 polypeptide subunits of respiratory chain complexes, along with the 22 tRNAs and 2 rRNAs (12S and 16S) (5). Mutation rates of mtDNA are generally 10- 100 times higher than those of nDNA (6) because mtDNA is compact (intron-less) and lacks an efficient DNA repair mechanism. It replicates rapidly by a unique D-loop mechanism without proofreading and it also lacks the protection of histones or DNA-binding proteins (7). Furthermore, mtDNA is attached, at least transiently to the mitochondrial inner membrane where ROS (reactive oxygen species) are generated as byproducts of OXPHOS in the ETC (8, 9). Several types of mtDNA point mutations and deletions have been identified in the affected tissues of patients with overt mitochondrial diseases (10-15). Large-scale deletions of mtDNA were first observed in the skeletal muscle of patients with mitochondrial myopathy (16). This type of DNA rearrangement has later been shown to occur frequently in the muscle of patients with chronic progressive external ophthalmoplegia (CPEO), Kearns-Sayre syndrome (KSS) and Pearson’s marrow-pancreas syndrome and other multisystemic disorders and male infertility (17, 18).

The accumulation of mtDNA with the common 4977 bp and 7436 bp large-scale deletions are well recognized to be associated with aging in various human tissues (19, 20). The 4977 bp deletion has been established to be the most common mtDNA mutation in affected tissues of about 40% of patients with mitochondrial myopathy (17, 18). Kao et al. first demonstrated the association of the 4977 bp deletion of mtDNA with low motility of the human spermatozoa. Several studies have also demonstrated that multiple mtDNA deletions are associated with defective sperm function and diminish fertility in men (14, 21-25). It has been suggested that these mutations cause infertility by affecting sperm motility. However, low levels of mtDNA deletions have been identified in human spermatozoa and studies have not found a clear relationship between large-scale mtDNA deletions and male infertility. Therefore, the identification of mtDNA mutations in the pathophysiology of human spermatozoa dysfunction is considered to be important better understanding the etiology of idiopathic infertility in men.

## Materials and Methods

### Study subjects and semen analysis

In this analytic study, a total of 25 semen samples were provided from the normozoospermic infertile patients ages 24-38 years attending the Infertility Clinic of the Fatemehzahra Hospital in Babol, Iran, in 2010. This study was conducted with the approval of the Medical Research Ethics Committee of the Faculty of Medical Sciences of Babol University. An informed written consent was obtained from all the subjects participating in the study. Individuals with a significant medical history, signs of defective androgenisation, testicular trauma, chromosomal disorders, cryptorchidism, vasectomy, endocrine disorders, leukocytospermic and, cigarette smoking and alcohol consumption were excluded from this study. The samples were collected into sterile containers after 3 days of abstinence and were allowed to liquefy at 37˚C for 30 minutes. Routine semen analysis was performed within 1 hour according to World Health Organization guidelines (26).

In order to remove much of the debris and contaminating leukocytes from the ejaculate and purify the spermatozoa according to motility, each sample underwent separation into two sections using the swimup method. Then, the 50 samples that were obtained from the swim-up method were classified into two groups, the normal motility group (including motile spermatozoa) and abnormal motility group (including immobile spermatozoa). After, the motility and morphology of the spermatozoa were assessed using microscopic examination. The morphology of the spermatozoa was reported according to Kruger,s criteria in which morphology <14% was considered abnormal (Table 1) (27).

**Table 1:** Comparison of sperm morphology and motility after swim up method in the study subjects


	Normal motility group(n=25)	Abnormal motility group(n=25)	P value

**Sperm morphology (%)**	25.66 ± 5.61	16.69 ± 7.02	<0.001
**Sperm motility (%)**	88.09 ± 4.58	49.78 ± 25.20	<0.001


Data are expressed as means ± SD. Comparison of mean values between both groups was performed with an independent t test. P<0.05 was considered statistically significant.

### Spermatozoa separation by swim-up procedure

After liquefaction, swim-up procedure was performed by adding 1 mL semen to the bottom of a Falcon tube (15 mL) containing 2 mL of fresh Ham’s F-10 medium (include 10% BSA; bovine serum albumin) using a sterile Pasteur pipette. The tubes were then placed in a 45˚ angle and incubated at 37˚C in 5% CO_2_ for 30 minutes. After the incubation period, ~1.0 ml of the supernatant including motile spermatozoa was collected as a normal motility sample and immobile spermatozoa under the tube were collected as an abnormal motility sample. The samples were then centrifuged at 330×g for 7 minutes. The supernatants were aspirated and the pellets re-suspended in 0.5 mL of Ham’s F-10.

### Preparation of human spermatozoa DNA

The total DNA of human spermatozoa was extracted according to the method of Kao et al. (23) with minor modifications. After centrifugation for 10 minutes at 2000×g at room temperature, the pellet of spermatozoa was washed twice with 0.9% NaCl solution and an aliquot of 2-3×10^7^ spermatozoa was incubated at 56˚C for 2 hours in a lysis buffer containing 2% sodium dodecyl sulphate (SDS), 10 mM dithiothreitol (DTT), 100 μg/mL proteinase K and 50 mM Tris-Cl (pH=8.3). After digestion, supernatants were extracted with phenol, followed by phenol/chloroform (1:1, v/v), and chloroform. DNA was precipitated with isopropanol (1:1, v/v) and one-tenth volume of 3 M sodium acetate (pH=5.6) and then incubated at -20˚C overnight. After washing with 75% ethanol (v/v), the pellet was dried and re-suspended in doubledistilled water and stored at -20˚C until use.

### Synthesis of oligonucleotide primers

Oligonucleotide primers encompassing the target DNA sequence were chemically synthesized by Isogen Life Science (Demeen, Netherlands). The nucleotide sequences and sizes of the polymerase chain reaction (PCR) products amplified from each of the primer pairs are shown in table 2. The LF1-HR1 and LF2-HR2 primer pairs were used for the amplification of 533 bp and 280 bp PCR products of total (deleted and wild-type mtDNA), respectively. The primer pairs LF3-HR4, LF4-HR4 and LF4-HR3 were used for the detection of the ~ 5 kb deleted mtDNA.

**Table 2:** Oligonucleotide primers used for PCR amplification of the 4866 and 4977 bp deletions in the mtDNA of human spermatozoa


Primer pair	Amplified position	Length of amplified PCR products (bp)		
	5´→3´	Normal mtDNA	4866 bp-deleted mtDNA	4977 bp-deleted mtDNA

**LF1-HR1^a^**	3304-3836	533	-	-
**LF2-HR2^a^**	5461-5740	280	-	-
**LF3-HR4^b^**	8161-14020	5860	994 bp	883 bp
**LF4-HR4^c^**	8251-14020	5770	904 bp	793 bp
**LF4-HR3^c^**	8251-13650	5400	534 bp	423 bp


^a^; The primer sets used for the determination of the total mtDNA , ^b^; The primer sets used for normal long-range PCR and ^c^; The primer sets used for the determination of the 4866 bp and 4977 bp-deleted mtDNA. LF1 (3304-3323) 5΄-AACATACCCATGGCCAACCT-3΄ LF2 (5461-5491) 5΄-CCCTTACCACGCTACTCCTA-3΄ LF3 (8161-8180) 5΄-CTACGGTCAATGCTCTGAAA-3΄ LF4 (8251-8270) 5΄-GCCCGTATTTACCCTATAGC-3΄ HR1 (3836-3817) 5΄-GGCAGGAGTAATCAGAGGTG-3΄ HR2 (5740-5721) 5΄-GGCGGGAGAAGTAGATTGAA-3΄ HR3 (13650-13631) 5΄-GGGGAAGCGAGGTTGACCTG-3΄ HR4 (14020-14001) 5΄-ATAGCTTTTCTAGTCAGGTT-3΄

### Long-range polymerase chain reaction

**Fig 1:**
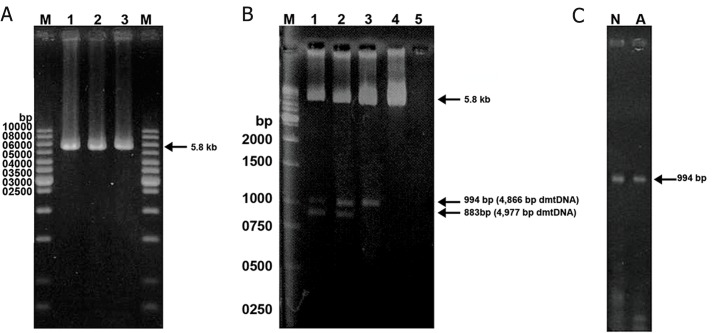
Detection of large-scale deletions of mtDNA from human washed sperm by long-range PCR method. A: The 5860 bp band represents the PCR product of normal mtDNA with primer pair LF3-HR4. Lane M is the 1-kb DNA size. B: Using the primer sets LF3-HR4, the 5860 bp band was amplified from the wild-type mtDNA, the 994 bp and 883 bands were amplified from the 4866 bp and 4977 bp-deleted mtDNA, respectively. Spermatozoa in lanes 1-4 had the motility scores of 5.0, 20.0, 30.0, 40.0% respectively. Lane 5 is the blank, in which the sperm DNA was omitted from the reaction mixture. Lane M is the1-kb DNA size marker. C. The arrow indicates the band of 994 bp produced with primer pair LF3-HR4. Using a short extension time of 1 minute at 72˚C, the longer DNA product from wild-type mtDNA could not be produced and only mtDNA with 4866 bp-deletion was amplified. Lanes N and A normal and abnormal groups, respectively.

To detect the common mtDNA deletion (4977 bp), a desired large segment of mtDNA (5.8 kb) was amplified from 20 ng of DNA in a 50 μl reaction mixture containing 200 μM of each dNTP, 0.5 μM of LF3 and HR4 primers (Fig 1, Table 2), 2 units of HLTaq DNA polymerase (Bioneer, Seoul, Korea), 40 mM KCl, 1.5 mM MgCl_2_ and 10 mM Tris-HCl, (pH=9.0) PCR was carried out for 35 cycles using the thermal profile of denaturation at 94˚C for 1 minute, annealing at 56˚C for 1 minute, and primer extension at 72˚C for 5 minutes. The PCR products were separated on 1% agarose gel electrophoresis, stained with ethidium bromide (1 μg/ml) and visualized by transillumination under UV light.

### Primer-shift PCR

In order to ascertain that an amplified DNA fragment was not due to mispriming in the presence of the large-scale deletions in mtDNA, we identified mtDNA deletions by primer-shift PCR (28) and using primer pairs LF4-HR4 and LF4-HR3 (Table 2).

### Semi-quantitative PCR

The proportion of the mtDNA with the 4866 bp deletion in each of the spermatozoa DNA samples was determined with a semi-quantitative PCR method (19). The total DNA of the spermatozoa was serially diluted twofold with distilled water. The primer pair LF2-HR2 was used for the amplification of a 280 bp DNA fragment from the total mtDNA and the primer pair LF4-HR3 was used for the amplification of a 534 bp PCR product from the mtDNA molecules with the 4866 bp deletion. Amplified DNA fragments were separated by electrophoresis on a 1.5% agarose gel. The proportion of mtDNA with the 4866 bp deletion was determined as the ratio of the highest-fold dilution that allowed the 534 bp PCR product to be visible on the stained gel to the dilution that allowed the 280 bp PCR product to be visibly amplified from the total mtDNA under identical conditions.

### DNA sequencing

The PCR product (534 bp mtDNA fragment) amplified from the 4866 bp deleted mtDNA using the LF4- HR3 primer pair was purified with the PCR product recovery kit (Roche Applied Science, Mannheim, Germany). Direct sequencing of purified PCR product was performed at Seq Lab (GOHingen, GmbH, Germany).

### Statistical Analysis

The data were expressed as mean ± SD. The mean values were compared using the independent t test with SPSS 11 for Windows software (SPSS Inc., Chicago, IL, USA). McNemar’s test was used to compare the frequency of mtDNA deletions between the two groups.In all cases, p<0.05 was considered statistically significant.

## Results

**Fig 2:**
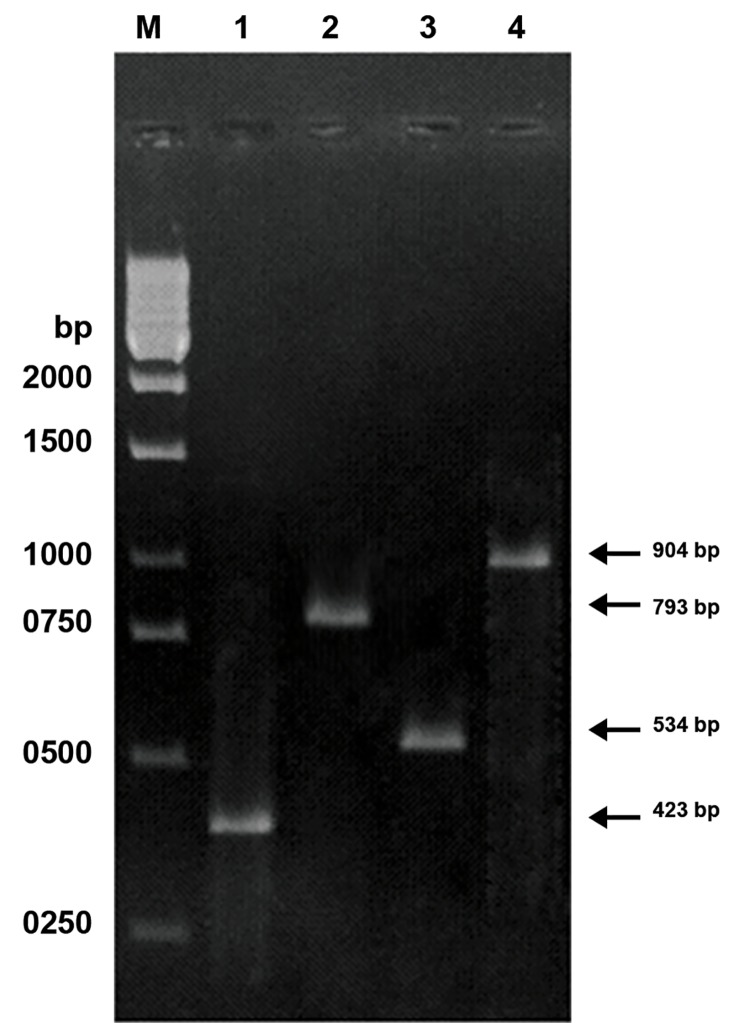
Detection of the 4866 and 4977 bp-deleted mtDNA by the primer shift PCR method in human spermatozoa. Lanes 1-2 represent the PCR products of 423 and 793 bp amplified from the 4977 bp-deleted mtDNA with primer pair LF4-HR3 and LF4-HR4, respectively. Lanes 3-4 represent the PCR products of 534 and 904 bp amplified from the 4866 bpdeleted mtDNA with primer pairs LF4-HR3 and LF4-HR4 respectively. Lane M indicates the 1-kb DNA size marker.

Based on standard semen analysis, motility and morphology of the spermatozoa after swim- up in normal motility group were significantly higher (p<0.001) in comparison with the abnormal motility group (Table 1). Using long-range PCR and primershift PCR techniques with the primer sets LF3-HR4, LF4-HR4 and LF4-HR3, we screened the existence of two large-scale deletions of mtDNA in human spermatozoa (Figes1, 2). The results of long-range PCR with the primer set LF3–HR4 revealed three bands with lengths of 5860 bp from the wild-type mtDNA, 994 bp and 883 bp from deleted mtDNA. The primer-shift PCR results clearly demonstrated a novel 4866 bp deletion along with the common 4977 bp-deleted mtDNA in the spermatozoa with different motilities (Fig 2). By using the primer pairs LF4-HR4 and LF4-HR3, PCR products of 904, and 534 bp from the 4866 bp-deleted mtDNA and, 793, 423 bp from the 4977 bp-deleted mtDNA were obtained, respectively (Table 2).

**Fig 3:**
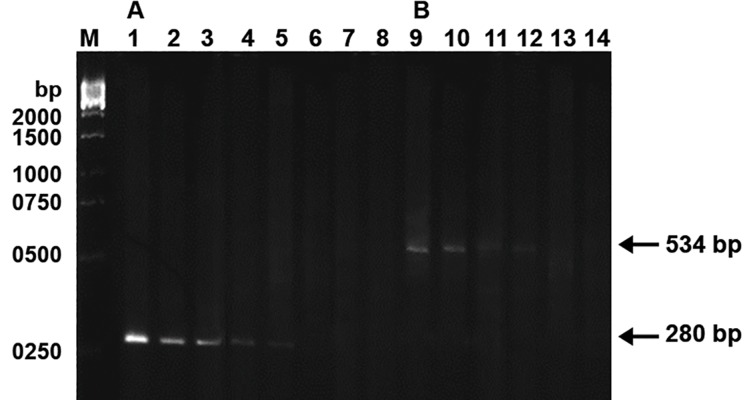
Semi-quantitative PCR analysis of mtDNA with the 4866 bp deletion using serial dilution method in human spermatozoa. A. Lanes 1-8 represent the PCR products amplified from total mtDNA serially diluted 2^8^, 2^9^, 2^10^, 2^11^, 2^12^, 2^13^, 2^14^, 2^15^-fold, respectively with primer pair LF2-HR2. B. Lanes 9-14 represent the PCR products amplified from 4866-bp deleted mtDNA serially diluted to 2^1^, 2^2^, 2^3^, 2^4^, 2^5^, 2^6^-fold, respectively with primer pair LF4–HR3. Lane M indicates the 1-kb DNA marker.

Direct sequencing of the 534 bp PCR product revealed that it was amplified from the mtDNA with a novel 4866 bp deletion. This deletion is located between nucleotide position (np) 8270 and np 13136 and flanked by a seven-nucleotide direct repeat of 5΄-ACCCCCT- 3΄ within the deleted area, between np 8271- 8277 and np 13127-13133 (Fig 3). DNA sequencing was also performed on a 432 bp PCR product from mtDNA. As expected, the analysis of the nucleotide sequences flanking the break points of the 4977 bp deletion revealed a 13 bp direct repeat (5΄-ACCTCCCTCACCA- 3΄) associated with this common deletion (data not shown). These two deletions were shown in both normal and abnormal motility groups. Also, 13 samples had both deletions of mtDNA (Table 3). The frequency of occurrence of mtDNA with the 4866 bp-deleted mtDNA (dmtDNA^4866^) and 4977 bp-deleted mtDNA (dmtDNA^4977^) was different in both groups. The abundance of the these deletions in abnormal motility group was 56% for dmtDNA^4866^ and 52% for dmtDNA^4977^ in comparison with normal motility group with 24% for dmtDNA^4866^ and 28% for dmtDNA^4977^, respectively (Table 3). Overall, the incidence of deleted mtDNA in the abnormal motility group was higher than the normal motility group.

**Table 3:** The occurrence of the 4866 and 4977 bp deletions of mtDNA in the spermatozoa with different motility after swim-up method in the two study groups


Samples	Abnormal motility group (motility ≤ 50%)		Normal motility group (motility ≥70%)	
	dmtDNA^4866^	dmtDNA^4977^	dmtDNA^4866^	dmtDNA^4977^

**1**	-	-	-	-
**2**	-	-	-	-
**3**	+	+	+	+
**4**	-	-	-	-
**5**	-	+	-	-
**6**	+	+	-	-
**7**	+	+	-	-
**8**	-	-	-	-
**9**	+	+	+	+
**10**	+	-	+	-
**11**	+	+	+	+
**12**	+	+	+	+
**13**	+	+	-	-
**14**	-	-	-	-
**15**	+	-	-	-
**16**	+	+	-	-
**17**	-	-	-	-
**18**	-	-	-	-
**19**	-	-	-	-
**20**	+	+	+	+
**21**	+	+	-	+
**22**	-	-	-	-
**23**	+	+	-	+
**24**	-	-	-	-
**25**	+	+	-	-
**Frequency (%)**	56*	52**	24*	28**


The spermatozoa with different motility were separated by swim-up method and divided to two groups. dmtDNA^4866^: 4866 bp deleted mtDNA, dmtDNA^4977^: 4977 bp deleted mtDNA. +; Presence of the indicated mtDNA deletion; -; Absence of the indicated mtDNA deletion, *; P value=0.008 and **; P value= 0.031.

## Discussion

Sperm motility is one of the most important factors of fertility in men (1, 2). Several studies have shown that an increase in the concentration of individual mitochondrial OXPHOS inhibitors results in a decline in sperm motility (29, 30). A correlation has been found between semen quality and the respiratory chain function in sperm mitochondria (30, 31). This appeared to be a relationship between mitochondrial DNA T-haplotype and poor sperm motility (30). Spiropoulos et al (13). reported that the high frequency of the A3243G mtDNA mutation strongly correlates with low sperm motility. Thangaraj et al. (32) identified two nucleotide deletions in the COII genes (at np 8195 and 8196) of sperm mtDNA, introducing a stop codon (AGA), which might be responsible for low sperm motility.

Kumar et al. (14) showed high frequency of some nucleotide changes in the mitochondrial genes including ATPase (6 and 8), ND (2, 3, 4 and 5) in the semen of the oligoasthenozoospermic (OA) infertile men. Pereira et al. (33) did not find any correlation between mutation C11994T in ND4 gene and low sperm motility in OA infertile men. It is believed that any defects or abnormal changes in the arrangement of mitochondrial DNA may affect sperm motility in idiopathic asthenozoospermic and OA patients (34). So far, multiple large-scale mtDNA deletions have been identified in the sperm of infertile men, especially in men with low sperm motility (1, 21-25). Some studies observed a negative relationship between the common 4977 bp mtDNA deletion and sperm motility (21, 25). Kao et al. (1) first observed a higher incidence of the common 4977-bp deletion in the mtDNA of lower Percoll-fractionated spermatozoa of patients with infertility or subfertility. They also identified presence of two novel deletions, of 7345 and 7599 bp in length in mtDNA of poor motile sperm (23).

In one study from a Northern Iranian population, the occurrence of the 4977 bp deletion of spermatozoa in infertile men with varicocele was significantly higher than in control healthy men (22). However, some studies have not found a direct correlation between the 4977 bp and 7.4-7.6 kb deletions, and low sperm motility (25) or for the 4977 bp deletion and semen quality (35). Although, they showed the persistence of multiple mtDNA deletions in both normozoospermic and oligoasthenoteratozoospermic men. Lestienne et al. revealed the presence of multiple mtDNA deletions in both spermatozoa and skeletal muscle in a patient with OA. They suggested that the multiple human mtDNA deletions might be of nuclear origin since at least three nuclear loci have been ascribed to multiple mtDNA deletions: 10 q 23.3-24.3, 3 p 14.1-21.2 and 4 p 16 (36).

**Fig 4:**
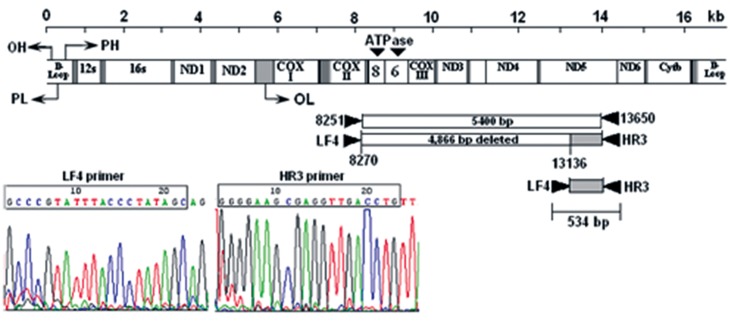
DNA sequence of the 534 bp PCR product was obtained by using the LF4-HR3 primer pair, indicating that 534 bp band was amplified from mtDNA with a novel 4866 bp deletion while the wild-type mtDNA yields a product of 5400 bp (8251-13650).

In the present study, we investigated correlation between large-scale deletions of the mtDNA with sperm motility. Our PCR analysis demonstrated a novel 4866 bp (Fig 4) and the common 4977 bp deletions from the mtDNA of spermatozoa (Fig 1). We also confirmed the persistence of these mtDNA deletions in both groups (Fig 2). Our results showed a higher incidence of mtDNA with the 4977 bp and 4866 bp deletions in abnormal motility group than in normal motility group. While we found the 4977 bp and a novel 4866 bp deleted mtDNA with primer pair LF4-HR3 (8251-13650), Kao et al. (1) only identified the 4977 bp mtDNA deletion in the human spermatozoa with this primer pair. Furthermore, Fahn et al. (37) also observed the 4977 bp deletion along with a novel 4839 bp deletion with the same primer set in lung tissue. It appears that one of the causes of differences in such mutations might be a reflection of the differences in tissues or populations. Therefore, it is important to note that some other mtDNA deletions may exist that have been undetected. Reynier et al. found that about 85% of sperm samples contained large-scale mtDNA deletions of variable sizes, and that most subjects had 2 to 7 deletions of mtDNA. They suggested that these mtDNA deletions are similar to those observed in skeletal muscle, myocardium, and other tissues of aged individuals (24). In our study, 13 of the samples had 2 large-scale mtDNA deletions. Ieremiadou and Rodakis showed that PCR slippage and primer mismatches to nDNA might lead to overestimates in the frequency of deletions (21). The large-scale deletions result in complete removal or truncation of some structural genes and tRNA genes of mtDNA. The defective protein subunits encoded by the deleted mtDNA are assembled with nDNA encoded subunits to yield impaired respiratory enzymes that may further enhance ROS or free radical production and result in a progressive decline in the bioenergetic function of mitochondria and hence low sperm motility. Thus, random attacks on the naked mtDNA by ROS or free radicals may cause mutations in the mtDNA with pathological consequences (2, 23). It is suggested that ROS elicited oxidative damage to DNA might be fixed as large-scale deletions of mtDNA in spermatozoa (2).

Furthermore, spermatozoa are especially susceptible to oxidative stress because their plasma membranes are rich in unsaturated fatty acids (38). One of the common oxidative byproducts of DNA, 8-hydroxy-2-deoxy guanosine (8-OHdG) was identified in the human spermatozoa. Also, the level of sperm 8-OHdG in infertile patients was significantly higher than the healthy subjects (39). However, the mechanisms on how these deletions are generated remain unclear, but two major hypotheses have been considered to generate these deletions: i. replication through slipped mispairing between two repeats and ii. repair mediated by mtDNA double-strand breaks (11). A predominance of rearranged molecules over wild-type, (heteroplasmy), or the persistence of mutated or deleted molecules only (homoplasmy), can result in the onset of mtDNA disease (4). Though energy requiring organs like brain, muscle and heart are mostly affected by heteroplsamy (10), such effect on mtDNA of the spermatozoa is not well studied. More studies are needed to understand the role of heteroplasmy in sperm mtDNA of infertile men, since homoplasmy mutant mtDNA has been found in OA infertile patients (40). According to our results, the frequency of the 4977 bp and a novel 4866 bp- deleted mtDNA in the abnormal motility group was higher than the normal motility group (p<0.05). Our results indicated that the difference of frequency between the two groups is nonrandom and suggest an association between mtDNA deletions and poor sperm motility, similar to the findings of Kao et al. (1, 23).

## Conclusion

We conclude that there is a direct correlation between large-scale mtDNA deletions and low sperm motility. These deletions might be an important factor for poor sperm motility especially in asthenoteratozoospermic patients, but we can not say certainly that declined fertility in men is associated with these deletions. Therefore, more studies are required with larger samples of diagnostically categorized infertile males. Furthermore, the identification of largescale deletions of mtDNA could be important to better understand the etiology of idiopathic infertility and treatment/ assisted reproductive techniques.
